# Bilateral Thalamic Ischemic Stroke Secondary to Occlusion of the Artery of Percheron

**DOI:** 10.7759/cureus.2676

**Published:** 2018-05-23

**Authors:** Miguel A Garcia-Grimshaw, Mariana Peschard-Franco, Francisco A Gutierrez-Manjarrez

**Affiliations:** 1 Internal Medicine, Hospital General De Tijuana, Universidad Autonoma De Baja California, Campus Mexicali, Tijuana, MEX; 2 School of Medicine, Instituto Tecnológico Y De Estudios Superiores De Monterrey, Monterrey, MEX; 3 Neurology, Hospital General De Tijuana, Tijuana, MEX

**Keywords:** percheron syndrome, artery of percheron, bilateral thalamic ischemic stroke, thalamic stroke, hypersomnolence, stroke

## Abstract

The occlusion of the artery of Percheron (AOP) is a rare condition that causes bilateral thalamic ischemic stroke with or without midbrain involvement. It happens as a result of an anatomical variant of the diencephalic irrigation, in which the thalamic paramedian arteries arise from a common trunk from the posterior cerebral artery (PCA), which generates a clinical syndrome characterized by bilateral vertical gaze palsy, memory impairment and hypersomnia. In this case, we report a 62-year-old woman admitted to the emergency room with altered mental status, mainly somnolence. On physical examination, she was somnolent, apathetic and with no motor deficit. Magnetic resonance imaging (MRI) of the brain demonstrated bilateral thalamic hyperintensities and midbrain involvement in diffusion-weighted imaging (DWI) and T2 sequences, suggesting occlusion of the AOP. Bilateral thalamic infarction due to this anatomical variant is an entity with a low prevalence, and its diagnosis can be delayed because of the wide spectrum of clinical signs.

## Introduction

The artery of Percheron (AOP) is an uncommon anatomical variant of the thalamic-mesencephalic artery that arises from the posterior cerebral artery (PCA) and supplies the medial region of both thalami. This anatomical variant was first described by the French neuropathologist Gerard Percheron in 1973. The occlusion of this artery should be considered in the differential diagnosis of bilateral thalamic lesions, in which the patient often presents with altered mental status, mainly somnolence. Worldwide, the occlusion of this artery is responsible for 0.1 to 2% of ischemic strokes [1]. This anatomical variant is estimated to exist in 4-11.7% of the general population [2]. Regarding predilection by sex or age, the samples reported are very different and it is considered that these variables depend on the etiology of the event [3]. This case details the history of a 62-year-old female patient with an established diagnosis of hypertension who presented with somnolence and speech alterations. The differential diagnosis included several feasible etiologies for the patient’s signs. She was eventually diagnosed with infarction of the AOP.

## Case presentation

**Figure 1:**
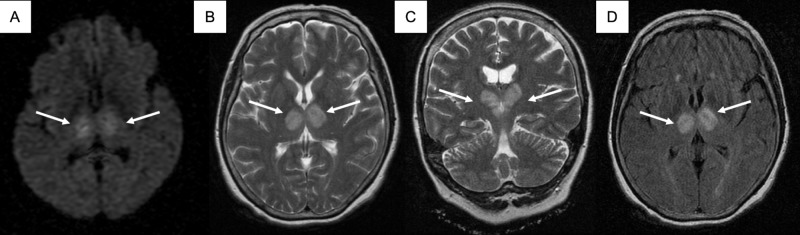
Case MRI images. (A) Axial MRI in DWI sequence showing bilateral thalamic restricted diffusion. (B-C) Axial and coronal section T2 sequence with bilateral thalamic hyperintensities with extension to the midbrain. (D) Axial FLAIR sequence in which bilateral thalamic hyperintensities are seen. DWI: Diffusion-weighted imaging; FLAIR: Fluid-attenuated inversion recovery; MRI: Magnetic resonance imaging.

A 62-year-old woman with a past medical history of hypertension is admitted to the emergency room due to altered mental status noticed on awakening. She was somnolent, bradycardic and hypertensive, with a heart rate of 50 beats/min and blood pressure of 165/82 mmHg. On the neurological exam, the patient had a Glasgow Coma Scale (GCS) of 12 points (ocular: three points, verbal: four points, motor: five points), the patient was apathetic with non-fluent speech and normal nomination; no other abnormalities were found. The laboratory workup at admission was normal. An emergency brain computed tomography (CT) showed bilateral thalamic hypodensities. A 12-lead electrocardiogram, chest X-ray, transthoracic echocardiogram, carotid and vertebral Doppler ultrasound were performed, all of them reported normal. Magnetic resonance imaging (MRI) of the brain showed bilateral thalamic hyperintensities in diffusion-weighted imaging (DWI), T2 and fluid-attenuated inversion recovery (FLAIR) sequences (Figure 1).

The etiology of the stroke remained cryptogenic after the approach. The patient was discharged after eight days with improvement in alertness. She persisted with episodes of somnolence, apathy, bradylalia and hypophonia without any motor deficit.

## Discussion

**Figure 2:**
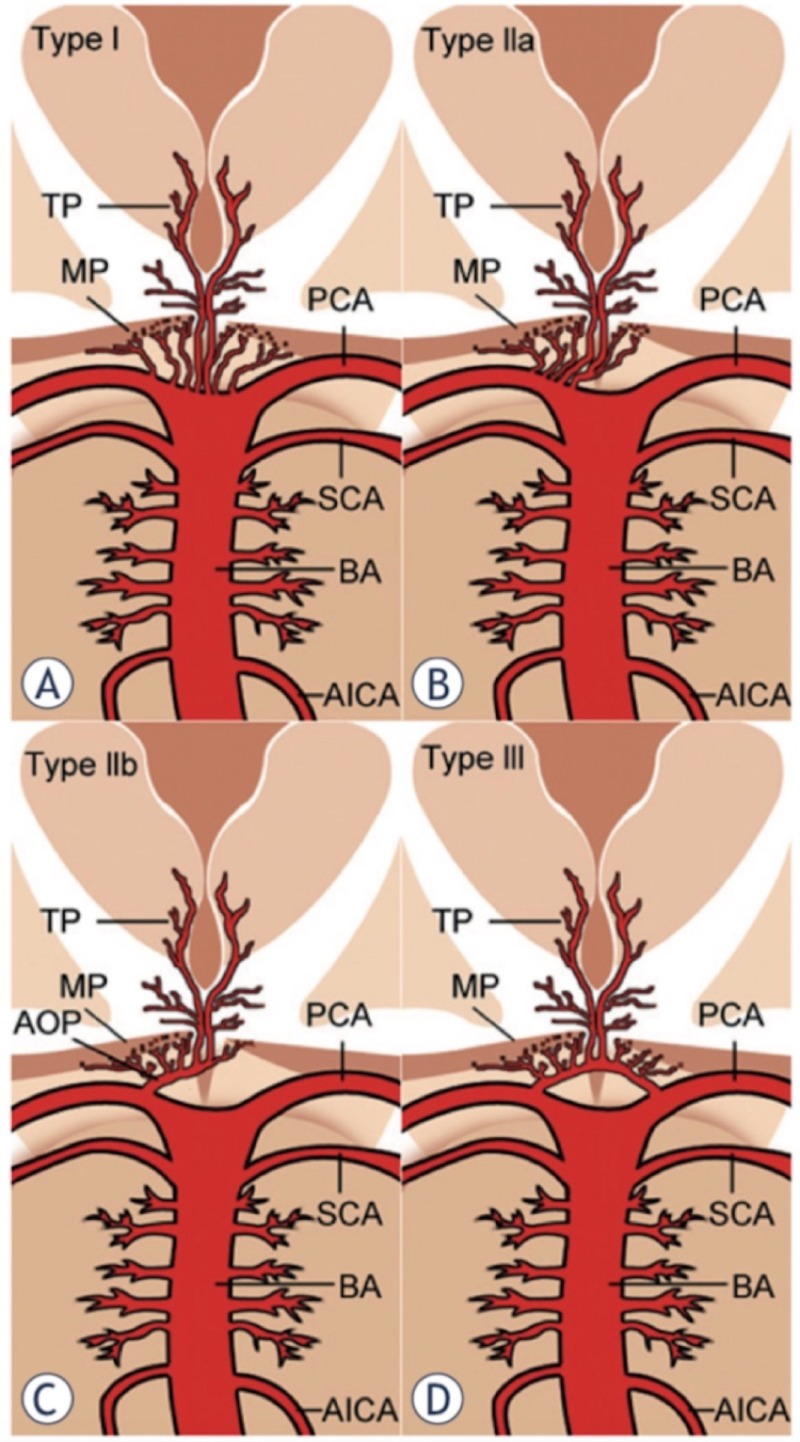
Anatomic variations of the arterial supply to the paramedian thalamic-mesencephalic region as described by Percheron. (A) Variant I, (B) variant IIa, (C) variant IIb “the artery of Percheron”, (D) variant III. Vessels marked by initials: thalamic perforators (TP), midbrain perforators (MP), posterior cerebral artery (PCA), superior cerebellar artery (SCA), basilar artery (BA), anterior inferior cerebellar artery (AICA) and artery of Percheron (AOP) [6].

The normal irrigation of the thalamus is supplied by branches emerging from the posterior communicating arteries (PcomA) and the P1 and P2 segments of the posterior cerebral artery (PCA). The irrigation of the thalamus is classically divided into four segments: anterior, paramedian, inferolateral and posterior. The anterior territory receives blood from the polar arteries, which arise from the PcomA; the paramedian territory is irrigated from the thalamo-perforating arteries which originate from segment P1 of the PCA; the inferolateral territory is supplied by the thalamo-geniculate arteries, which emerge from the P2 segment of the PCA; and the posterior territory is supplied by the posterior choroidal arteries, which arise from the P2 segment of the PCA. There are multiple anatomical variables of the vasculature of this territory, in particular, the ones of the polar artery; there have been described up to eight variants of this artery [4]. It was not until 1973 when the French neuropathologist Gerard Percheron described the four main variants of the thalamic irrigation and the artery that bears his name: “The artery of Percheron”. This is a rare anatomical variant that forms a single thalamic-perforating branch (Figure 2). The occlusion of this artery leads to the characteristic findings of bilateral thalamic paramedian ischemia with or without midbrain involvement [5-6].

The risk factors of the artery of Percheron occlusion are similar to the ones of an ischemic stroke: small vessel disease (33-38.9%); cardioembolic source (0-22%); large vessel disease (13.2-22.2%); other causes like vasospasm secondary to subarachnoid hemorrhage, hemodynamic alterations, vascular dissection, distal ischemia of the PCA, aneurysms of the basilar artery, hypercoagulable states and vasculitis secondary to infections of the central nervous system (13-15.7%); and idiopathic causes (10%) [1, 7-8].

As result of the complexity of the thalamus function and anatomy, specially its irrigational variations previously mentioned, the clinical syndromes of these patients can differ depending on the sub-structure of the thalamus involved. There has been described an overlap with mesencephalic syndromes. The classic syndrome of occlusion of the AOP presents with bilateral vertical gaze palsy in 65%, memory impairment (anterograde and retrograde amnesic syndrome) in 58%, confusion in 53% and coma in 42% of the patients [9].

The onset of altered mental status can range from immediate to progressive over hours in 40% and 60% of the cases, respectively [8]. The rest of the symptoms comprise partial disorientation, confusion, hypersomnia with variable GCS with an average of eight points (ranging from 4-15 points), akinetic mutism, and emotional central paralysis. Speech alterations like dysarthria, Guberman and Struss aphasia, associated with hypophonia and dysprosody, reduced verbal fluency, preserved syntactic structure, but marked simplification of syntax, normal repetition, but occasional paraphasic errors also have been found. Other neurologic symptoms include apraxia, dysgraphia, miosis, photophobia, asterixis, loss of convergence, and ataxic gait. Neuropsychiatric changes like apathy and aggressiveness have been observed. There is a report of two cases in which the diagnosis was confused with conversion disorders [7, 10-12].

Brain MRI is the most specific imaging study for the diagnosis. The DWI sequence has a sensitivity of 100%, compared to CT scan which appears normal 50% of the time, making the MRI the best tool to establish an opportune diagnosis and the possibility to offer thrombolytic therapy [7]. Normally a hyperintense signal is observed in the T2 or FLAIR sequences with or without restricted diffusion and occasional enhancement specifically in the region that corresponds to the vascular territory of the AOP. The presence of the "V" sign has been described as a hyperintensity in axial sections in the FLAIR sequence or DWI along the pial surface of the midbrain, adjacent to the interpeduncular fossa with a sensitivity of up to 67% in patients with midbrain involvement. In a series of 37 cases, the topographic imaging patterns were symmetrical in 68% of the patients and asymmetric in 32%. Rostral midbrain involvement was observed in 57% of the patients. Each case was found to fit into one of four patterns: bilateral paramedian thalamic with midbrain (43%), bilateral paramedian thalamic without midbrain (38%), bilateral paramedian thalamic with anterior thalamus and midbrain (14%), and bilateral paramedian thalamic with anterior thalamus without midbrain (5%). Simultaneous infarctions were found in the cerebellum in 19% of the cases, as well as in the occipital lobe in 11%, and 14% in the territory of the middle cerebral artery. The angiographic study had very low sensitivity in this series, demonstrating only in one patient the anatomical variant of Percheron, coinciding with the low sensitivity of angiography reported by other authors. This concludes that the diagnosis is made upon clinical suspicion [4].

The differential diagnosis of bilateral thalamic lesions based on the clinical and imaging findings is listed in Table 1 [3, 13-14].

**Table 1:** Differential diagnosis of bilateral thalamic lesions.

Cause	Etiology
Vascular	Subarachnoid hemorrhage
	Thrombosis of the vein of Galen
	Top of the basilar syndrome
	Posterior reversible encephalopathy
	Infectious vasculitis
Infectious	Western Nile encephalitis
	Japanese encephalitis
Metabolic	Korsakoff encephalopathy
	Osmotic myelinolysis
	Fabry disease
	Fahr syndrome
Other	Creutzfeldt-Jakob disease
	Fatal familial insomnia
	Bilateral thalamic gliomas
	Leigh syndrome

The treatment is similar to the management of an ischemic stroke in another region of the brain, which consists of platelet antiaggregation therapy, and blood pressure and glycemic control. Thrombolysis would be appropriate if the patient arrives in a therapeutic window and qualifies for this management. There is a case report where selective endovascular thrombolytic therapy for the AOP occlusion showed clinical and imaging improvement after 24 hours [15].

The long-term prognosis of bilateral paramedian thalamic infarcts continues to be unknown. A series of 16 cases with paramedian infarction, reported persistent memory dysfunction and apathy in 13 patients, and impairment of the alertness in eight patients in their follow-up [3]. Another series of 18 patients reported that the life and function prognosis of these patients was favorable during the first three months; 61% of them having a favorable outcome with functional recovery performing their normal daily life activities [7].

## Conclusions

Bilateral thalamic infarction due to occlusion of the anatomical variant of Percheron artery is a rare entity with a low frequency, which can be difficult to identify because of its broad spectrum of clinical signs. It is noteworthy that the risk factors are similar to the ones of an ischemic stroke, and the initial etiological approach must be the same as with any stroke affecting other structure of the brain. In our patient, we found some of the classic clinical characteristics mentioned in the literature, including hypersomnolence, bradylalia, and apathy. The most remarkable signs mentioned by other authors are upper gaze palsy, impairment in memory and confusion. It is essential to get in mind that the most sensitive imaging study for this purpose is the MRI. The relevance of reporting this case is to acknowledge that the occlusion of this artery is an uncommon stroke syndrome, which can mimic other diseases, delaying its diagnosis.
